# Significance of IgG optical density ratios (index value) in single reactive anti-Dengue virus IgG capture ELISA

**Published:** 2016-12

**Authors:** Shiv Sekhar Chatterjee, Ankush Sharma, Shilpee Choudhury, Sushil Kumar Chumber, Mandeep Kaur, Ras Bage, Nittin Parkhe, Uma Khanduri

**Affiliations:** St Stephen Hospital, Delhi, India

**Keywords:** Dengue fever, IgG capture ELISA, Optical density ratio, Index value

## Abstract

**Background and Objectives:**

A single reactive IgG anti-Dengue virus ELISA test in the absence of IgM antibodies or NS1 antigen may denote current infection or past exposure to the virus. To determine whether IgG index value can be used to identify true current dengue infection we conducted a prospective observational study.

**Materials and Methods:**

Suspected dengue patients (n =1745) were tested in their first specimen by MAC-ELISA, GAC-ELISA and NS1 antigen ELISA. Patients with MAC-ELISA and NS1Antigen non-reactive but GAC-ELISA reactive results (n =57) in their first test were followed up and repeated sampling was asked for IgG index values were calculated according to the manufacturer’s instruction and classified as: low (2.2–2.5), medium (2.5–4.0) and high (>4.0).

**Results:**

16 out of 57 patients (28.1%) had low IgG Index value whereas 26 cases (45.6%) were categorized as medium and 15(26.3%) were classified as patients with high IgG index. Nine patients with paired reactive serology or antigen positive status were categorised as serologically confirmed dengue fever, 11 patients as not dengue with categorical evidence of other infections while the rest 37 casas with clinical, radiological and laboratory parameters suggestive of dengue but no serological confirmation as possible dengue. Among confirmed, possible and non-Dengue cases, 33.3, 32.4 and 0.0% had high Index value in comparison with 22.2, 29.7 and 27.3% showing low Index values, respectively.

**Conclusion:**

Our results suggested a high IgG response in favour of true dengue infection than past exposure while no conclusions should drawn from a low or medium reactive GAC-ELISA results in the absence of IgM antibodies and NS1 Ag.

## INTRODUCTION

Dengue fever, a fearsome arboviral infection, has the highest burden in South Asia and is especially menacing in large Indian metropolises (1). Specific diagnosis of dengue fever is based on antigen testing, serology, costly molecular methods (RT-PCR) and prohibitively expensive and laborious virus isolation (2, 3). NS1 antigen (NS1 Ag) test and IgM capture ELISA (MAC-ELISA) are the most common modalities of diagnosis in clinical practice (2). A reactive NS1 Ag test occurs in dengue patients during days 1–9 of fever occurrence but it has a poor sensitivity (4–7) and is also sometimes non-specific (6, 7). NS1 Ag may be undetectable especially in secondary dengue cases when anti-NS1 antibodies form immune complexes and hinder their detection (4, 7). Furthermore, a single reactive IgM or IgG anti-Dengue virus test is only suggestive for dengue infection (2, 7, 8). False diagnosis of dengue may be due to the persisting IgM antibodies originated from a previous infection (2, 8). Most authorities agree that IgM anti-Dengue antibodies usually persist for 2–6 months (6–11) with a median time period of 179 days for primary and 139 days for secondary infections (10). However this response is not uniform and IgM may disappear early in some cases (10).

The use of serum anti-Dengue IgG antibodies lies in demonstration of a four-fold rise in antibody titer in paired serum samples (2, 3, 8). Anti-dengue virus IgG starts rising during 5–7 days after primary infection and even earlier in secondary infections (9, 11, 12) and the highest IgG titers appear during the 3^rd^ week of a primary infection. In secondary dengue infections, IgG titers rise to extremely high levels (much higher than in primary infections) from the day 7 of fever to the next two weeks (9, 11). Anti-Dengue IgG antibodies persist for a long time even up to years (3, 12). Neutralization assays for detection of IgG antibodies are prohibitive in terms of cost and manpower for routine laboratories (2). ELISA formats are usually preferred where a rise of IgG titer can be estimated from a significant rise in the optical density (OD) values in paired samples. However, in many laboratories only one serum sample is received for Dengue antibody testing (6, 13). WHO recommends that a confirmatory acute dengue diagnosis by serological means needs IgM seroconversion or a four-fold increase of IgG antibody titers in paired sera (3). Development of anti-dengue IgG antibodies along with increased IgM is known to occur in secondary dengue infections (1, 3). In a group of secondary dengue patients, the IgM response will transiently develop or occurs later than IgG antibodies (2, 11). In those patients who are IgG sero-reactive the otherwise reliable NS1 antigen test has a poorer sensitivity than usual (1, 4, 7). The above two facts combine to result in a non-reactive IgM and NS1Ag test with a reactive IgG result leaves the diagnosis uncertain. This is because a single reactive anti-dengue IgG capture ELISA test (GAC-ELISA) in the absence of IgM antibodies or NS1 Ag may denote current infection or past exposure to the virus (2, 3, 8). In the present study, we focussed exclusively on patients with reactive anti-dengue virus IgG test in the absence of IgM antibodies or NS1 antigen.

## MATERIALS AND METHODS

A prospective observational study was carried out at St Stephen’s Hospital, Delhi from 2012 to 2014 (including two outbreak periods) to evaluate the utility of IgG reactivity in patients showing negative results in NS1 Ag and MAC-ELISA. All suspected dengue patients (n=1745; 958 males, 787 females) were tested for NS1 Ag, anti-dengue IgM and IgG via ELISA.

### Sample Collection

Clotted venous blood samples were collected in gel vacuettes and serum was separated by centrifugation at 1000 g for 5 min. Separated sera were tested immediately or stored at 4°C for maximum 18 hours before testing.

### ELISA tests

Both IgG and IgM ELISA tests were performed on a fully automated EVOLIS microplate system (Bio-Rad, USA). Antibody testing was done with Panbio Dengue IgM capture ELISA (Inverness Medical Innovations, Australia) and Dengue IgG capture ELISA (Inverness Medical Innovations Australia) kits. One negative control, one reactive control and calibrators in triplicate format were used in each run according to the manufacturer’s instructions. Cut-off value (COV) of each run was the product of the mean calibrator OD and calibration factor given in each kit. Index value (IV) (ratio of sample OD/COV) was calculated for each sample to determine results as reactive (IV > 2.2), negative (IV < 1.8) and equivocal (IV: 1.8–2.2) (10) according to the instructions.

To study the significance of antibody response level, index value of IgG positive samples was further classified as: low (2.2–2.5), medium (2.5–4.0) and high (>4.0). Any initial equivocal result was rechecked to confirm the results. NS1 Ag testing was done with Platelia^TM^ Dengue sandwich ELISA (Bio-Rad). One Negative control, two calibrators, and one positive control samples were used in each run and the cut-off value was calculated as mean of calibrators.

Admitted patients with reactive GAC-ELISA but non-reactive MAC and NS1 Ag-ELISA test results in their first samples were included in the study. Repeated sampling for paired dengue serology tests were asked from the corresponding physicians and only 14 repeated samples were collected. Meticulous clinical examination was carried out daily and all patients underwent the following investigations as per clinical requirement: complete blood count (CBC), blood culture, urine culture, thin and thick smears for malaria parasite, malaria antigen detection (Microgen Pvt Ltd, pan-pLDH detection), IgM anti-Leptospira antibody ELISA, IgM anti-Chikungunya antibody ELISA, liver function tests, renal function tests, chest X-ray and ultrasonography. CBC was done daily for the first 4 days of hospitalization and whenever required based on the clinical situation. Tachypnea, chest retractions, decreased breath sounds and decreased vocal resonance were considered as signs of pleural effusion. Abdominal distension with fullness of the flanks and presence of shifting dullness or fluid thrill was considered as ascites. Percentage of hemoconcentration was quantified by taking a difference between the maximum hematocrit at admission or anytime during hospitalization and the minimum hematocrit recording at convalescence or discharge (14). Dengue Hemorrhagic Fever was diagnosed according to WHO guidelines (3). Clinical presentation, clinical evidence of plasma leakage, laboratory tests (raised alanine transaminase and aspartate transaminase within 2–3 times normal limits, mildly decreased total protein and albumin), radiological evidence of plasma leakage and reactive gall bladder wall changes on ultrasonography were considered by the clinician before categorising these only IgG-Dengue-positive patients as possible dengue fever. A subset of these patients underwent repeated sampling and those showing seroconversion to IgM positive status or NS1 Ag positive status were categorised as serologically confirmed dengue fever. Patients with categorical evidence of other causes of fever like malaria, blood culture positive sepsis, culture positive urinary tract infection (UTI) or culture positive respiratory tract infection and those with none of the usual clinical, laboratory and radiological findings of dengue fever mentioned above were classified as non-Dengue fever.

### Statistical analysis

Statistical analysis was carried out in Microsoft Excel using Welch’s t-test to study the significance of differences.

## RESULTS

**Fig. 1.**
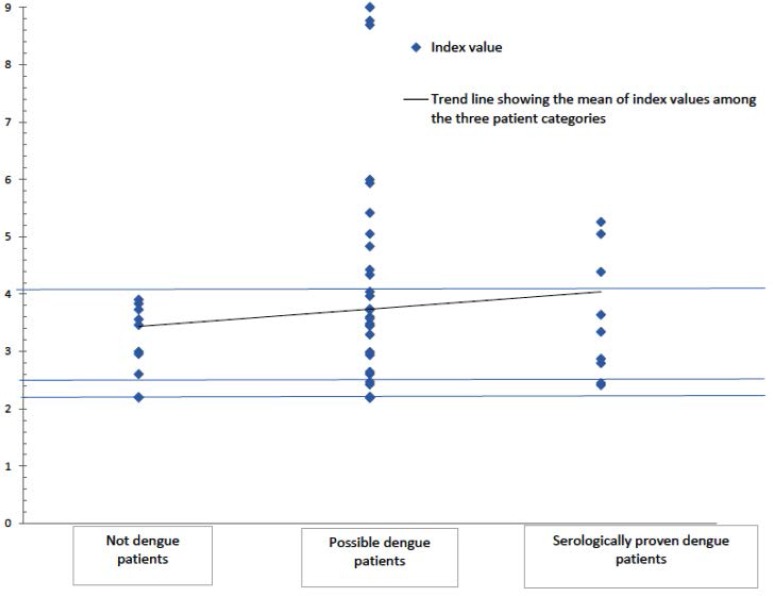
IgG index values in patients with non-reactive MAC ELISA, non-reactive NS1 Ag and reactive GAC-ELISA

Overall, 1198 patients including 745 males (62.2%) and 453 females (37.8%) in the outbreaks of 2012 and 2013 were reactive in at least one test (Table 1). Fifty seven patients were only GAC-ELISA reactive representing 16 low (28.1%), 26 medium (45.6%) and 15 high (26.3%) IgG index values (Table 2). All 57 patients had IgM/IgG OD ratio < 1.2. After an average duration of 5.2 ± 0.5 days of initial sampling, 14 patients were rechecked through serology and NS1 Ag; 7 samples represented reactive seroconversion to anti-Dengue IgM, and 2 cases became positive for NS1 Ag. These 9 cases with paired reactive serology test or antigen positive status were categorised as serologically confirmed dengue fever. Eleven patients were categorized as not dengue cases. These included two patients with lower respiratory tract infections, two *Escherichia coli* UTI, one *Plasmodium vivax* malaria, one *Escherichia coli* sepsis with UTI, one culture negative Liver abscess with *Escherichia coli* UTI, one autoimmune haemolytic anaemia with chronic renal failure, one bilateral pleural effusion of unknown etiology with progressive liver dysfunction, one acute vestibular neuronitis with one day fever of unknown etiology and one patient of diabetes mellitus type 2 with fever and cough of unknown etiology. The rest 37 patients were classified as possible dengue fever. Among confirmed dengue cases, possible dengue cases and non-dengue cases, 33.3, 32.4 and 0% had high index values, respectively compared to 22.2, 29.7 and 27.3% with low index values (Table 2, Fig. 1).

**Table 1.** Distribution of reactive dengue serology and antigen detection tests

**Tests**	**Reactivity in 2012 (%)**	**Reactivity in 2013 (%)**	**Overall reactivity (%)**
IgM anti-Dengue capture ELISA	530(76.9)	409(80.4)	939(78.4)
IgG anti-Dengue capture ELISA	433(62.8)	339(66.6)	772(64.4)
NS1 Antigen ELISA	415(60.2%)	288(56.6%)	703(58.7)
One of the above three kits	689	509	1198

**Table 2.** Anti-Dengue IgG ELISA index value in serologically confirmed, possible and non-Dengue patients

**Categories**	**No. IgG confirmed cases (%)**	**No. Serologically confirmed Dengue cases (%)**	**No. Possible Dengue cases (%)**	**No. Non-Dengue cases (%)**
Low (2.2–2.5)	16(28.1)	2(22.2)	11(29.7)	3(27.3)
Medium (2.5–4.0)	26(45.6)	4(44.4)	14(37.8)	8(72.7)
High (>4.0)	15(26.3)	3(33.3)	12(32.4)	0*

* *P* value = 0.04

## DISCUSSION

Anti-dengue IgG antibody response with IgM rise is known to occur in secondary dengue infections (1, 3). In a fraction of these secondary dengue patients, the IgM response is only transient or occurs after IgG. In these cases, usually just an IgG response will be documented (2, 11). A confounding observation in IgG sero-reactive patients is that the otherwise reliable NS1 Ag has a weak sensitivity (1, 4, 7). Anti-Dengue IgM antibodies generally persist for 2–6 months (3, 9–11) whereas anti-Dengue IgG antibodies appear after IgM approximately at day 7 of the fever (3, 7, 11, 12) in primary dengue infections but sometimes even as late as day 18 of hospitalization (6) and persists for a long time (7, 11). In secondary dengue infections IgG rises even earlier than in primary dengue within the first few days of fever (9, 11). Even healthy people in endemic areas may have detectable serum IgG antibodies against Dengue virus (1, 15, 16). The picture of only IgG reactive status can occur in a patient with past dengue but who currently has some other fever, like enteric fever, UTI, or other flaviviral infections (prolonged persistence of IgG and anamnaestic responses) (2, 7, 16, 17). False positive anti-Dengue IgG test results have been documented in patients with bacteremia, leptospirosis, Q fever, and other viral infections like Chikungunya, Tick-borne encephalitis, varicella, cytomegalovirus, and Epstein-Barr infections (16, 17). Separating the two groups, one with true dengue infection and the other with persistent (anamnestic) IgG or cross-reactive IgG requires neutralization test on paired serum samples or viral culture studies which are unavailable in most hospitals in developing countries (7, 12, 17). We undertook this study on the premise that these two groups of patients would have different IgG index valules. Note should be made that IgM index values have been used to design an efficient algorithm for identification of false-positive reactions wherein index values above 6.00 and 3.00 have been shown to reduce false positivity to 0 and 0.19%, respectively (18).

Compared to those with serologically confirmed dengue and possible dengue fever cases, none of non-Dengue patients had high IV (*P* < 0.05). However, results were non-contributory in those with low IV. Our findings suggest that a high IgG response is in favour of a true dengue infection rather than past exposure while no conclusions should be drawn from a low or medium reactive GAC-ELISA result in the absence of IgM antibodies and NS1 Ag. Repeated serological and NS1 Ag tests after a period of 7–10 days in such patients is suggested. Despite our requests, repeated sampling and testing was done in only 14 patients leading to confirmatory diagnosis of 9 patients. Another limitation of our study was the small sample size of IgG reactive only patients (n= 57) mainly due to the less common occurrence of such test results compared to other studies (15).
